# Light might suppress both types of sound‐evoked antipredator flight in moths

**DOI:** 10.1002/ece3.6904

**Published:** 2020-11-02

**Authors:** Theresa Hügel, Holger R. Goerlitz

**Affiliations:** ^1^ Acoustic and Functional Ecology Max Planck Institute for Ornithology Seewiesen Germany

**Keywords:** ALAN, Chiroptera, Lepidoptera, Noctuidae, playback experiment

## Abstract

Urbanization exposes wild animals to increased levels of light, affecting particularly nocturnal animals. Artificial light at night might shift the balance of predator–prey interactions, for example, of nocturnal echolocating bats and eared moths. Moths exposed to light show less last‐ditch maneuvers in response to attacking close‐by bats. In contrast, the extent to which negative phonotaxis, moths’ first line of defense against distant bats, is affected by light is unclear. Here, we aimed to quantify the overall effect of light on both types of sound‐evoked antipredator flight, last‐ditch maneuvers and negative phonotaxis. We caught moths at two light traps, which were alternately equipped with loudspeakers that presented ultrasonic playbacks to simulate hunting bats. The light field was omnidirectional to attract moths equally from all directions. In contrast, the sound field was directional and thus, depending on the moth's approach direction, elicited either only negative phonotaxis, or negative phonotaxis and last‐ditch maneuvers. We did not observe an effect of sound playback on the number of caught moths, suggesting that light might suppress both types of antipredator flight, as either type would have caused a decline in the number of caught moths. As control, we confirmed that our playback was able to elicit evasive flight in moths in a dark flight room. Showing no effect of a treatment, however, is difficult. We discuss potential alternative explanations for our results, and call for further studies to investigate how light interferes with animal behavior.

## Graphical abstract

Artificial light by humans negatively affects nocturnal wildlife. Here, we show that light might reduce both types of evasive flight behavior of moths, making them more vulnerable to attacks by bats.

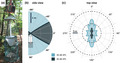

## 1 INTRODUCTION

Light pollution by artificial light at night (ALAN) has increased substantially over the last decades (Falchi et al., 2016; Fouquet, 2006; Hölker et al., 2010), adversely affecting plants and animals (Davies & Smyth, 2018; Knop et al., 2017; Longcore & Rich, 2004). The effects of light range from single individual's orientation, reproduction, and communication (Longcore & Rich, 2004) to whole communities, for example, by shifting the balance of predator–prey interactions (Bailey et al., 2019; Davies et al., 2013, 2014; Miller et al., 2017; Russo et al., 2019; Yurk & Trites, 2000). Echolocating bats and eared moths constitute a globally occurring predator–prey system of high ecological relevance (Boyles et al., 2011; Kasso & Balakrishnan, 2013; Kunz et al., 2011; Van Toor et al., 2019). Their interactions take place in the darkness of the night and are exclusively mediated by sound. Echolocating bats hunt by emitting ultrasonic calls (Fenton, 2003; Schnitzler et al., 2003), which eared moths can hear and react to with evasive flight (ter Hofstede & Ratcliffe, 2016; Roeder, 1962). Evasive flight likely consists of two stages: negative phonotaxis to fly away from distant bats (stage 1), and last‐ditch evasive maneuvers such as erratic flight or (power) dives to escape nearby attacking bats (stage 2). Corresponding to the differences in bat distance, negative phonotaxis is elicited at received sound pressure levels that are about 20 dB fainter than those that elicit last‐ditch maneuvers (Agee, 1969; Roeder, 1962, 1964, 1967).

Artificial light at night is of increasing concern for both bats and moths. While some bats may profit from exploiting prey accumulated at lights (Cravens et al., 2018; Rydell, 1992), other species are negatively affected while foraging, commuting, and roosting (Mathews et al., 2015; Stone et al., 2009, 2015; Straka et al., 2016, 2020). Moths are strongly attracted to light sources, leading to shortened foraging time (van Langevelde et al., 2017; Macgregor et al., 2019), disrupted navigation (Owens & Lewis, 2018), reduced pollination (Macgregor et al., 2017), and population decline (van Langevelde et al., 2017; Wilson et al., 2018). Furthermore, light increases the predation risk of moths, for two reasons. The accumulations of moths around light sources attract bats, thereby increasing the predation pressure on moths (Cravens et al., 2018; Rydell, 1992). In addition, light interferes with the moths’ sound‐evoked antipredator evasive flight response. In one set of studies, the sound‐evoked evasive flight of moths was compared under lit and unlit conditions, showing that light reduces the evasive flight. Wakefield et al. (2015) showed that only 24% of moths performed last‐ditch power dives under LED illumination compared with 60% of moths in darkness; that is, the light inhibited last‐ditch maneuvers in 60% of the moths that would react in darkness. Similarly, Svensson and Rydell (1998) reported that moths within a radius of 1 m around a light source showed ~ 60% less last‐ditch maneuvers than moths in darkness (where 100% of moths reacted). Finally, Minnaar et al. (2015) reported the most extreme effect: In darkness, bat diet was best explained by a model that included evasive flight of moths. In contrast, with light, bat diet was best explained by a model that incorporated a 100% reduction in moth evasive flight, suggesting that the light completely inhibited both stages of evasive flight (negative phonotaxis and last‐ditch maneuvers). In another set of studies, light exposure was kept constant while the sound received by the moth was manipulated. Those results showed that moths still exhibited some degree of evasive flight under illumination. Acharya & Fenton (1999) compared last‐ditch maneuvers in eared and deafened moths under illumination, showing that 48% of eared moths exhibited last‐ditch maneuvers when preyed on by bats, whereas deafened moths did not. Treat (1962) and Agee and Webb (1969) compared the number of caught moths at light traps with and without ultrasonic stimuli. Depending on sound stimulus and moth species, ultrasound playback reduced captures by 8%–49% (nine tympanate moth families with at least 39 caught individuals, Treat, 1962) and by 51%–86% (in *Heliothis zea*, Noctuidae, Agee & Webb, 1969) compared to the captures at the silent trap.

In summary, the first set of studies shows that light suppresses the sound‐triggered evasive flight in 60%–100% of the moths that would react in darkness. Contrasting this, the second set of studies shows that even in light, sound can still trigger evasive flight in 8%–86% of the moths. Noteworthy, these studies either only reported effects of light on last‐ditch maneuvers (Svensson & Rydell, 1998; Wakefield et al., 2015), or the results can be sufficiently explained by effects of light on last‐ditch maneuvers, as all moths had to fly through fields of high sound pressure level before entering the light trap (Agee & Webb, 1969; Treat, 1962). Only the modeling results of Minnaar et al. (2015) suggest that light completely suppresses both evasive flight responses. Therefore, while several lines of evidence suggest that last‐ditch maneuvers are suppressed by light pollution (with variable effect sizes), we lack a similar understanding of the effect of light on negative phonotaxis, and thus on the overall effect of light on evasive flight in moths. If the light‐induced suppression of negative phonotaxis is as strong as for last‐ditch maneuvers, this will strongly affect the predator–prey interactions between bats and moths, because negative phonotaxis is elicited over larger distances and larger spatial volumes than last‐ditch maneuvers. Here, we advanced the light‐trap approach of Treat (1962) and Agee and Webb (1969) to investigate the effects of light on both stages of evasive flight, negative phonotaxis and last‐ditch maneuvers. We combined the omnidirectional light field of light traps (attracting moths equally from all directions) with a directional ultrasonic playback that should elicit different stages of evasive flight depending on each moth's approach direction. As a moth approaches the light trap, its received light level will gradually increase independently from the approach direction, while its received sound pressure level will increase to different maximum values depending on the approach direction. Therefore, moths will be exposed to various combinations of light and sound pressure levels, covering a range of predator–prey scenarios (distant and close‐by bats) and light levels (distant and close‐by light sources) that a moth might encounter during the course of a night, allowing us to test the overall effect of light on both stages of evasive flight in moths. We compared moth captures at the light traps with and without acoustic playback, to measure the overall effect of light on the sound‐evoked evasive flight. In line with Minaar et al. (2015) who suggest that light suppresses both stages of evasive flight, we predicted equal moth counts at both light traps, as either stage of evasive flight would cause a decline in the number of caught moths. In contrast, if negative phonotaxis (stage 1) was not suppressed or both stages were only partially suppressed, we predicted lower moth counts at the ultrasonic than the silent trap.

## 2 METHODS

### 2.1 Setup, study site, moth capture, and measurement

**Figure 1:**
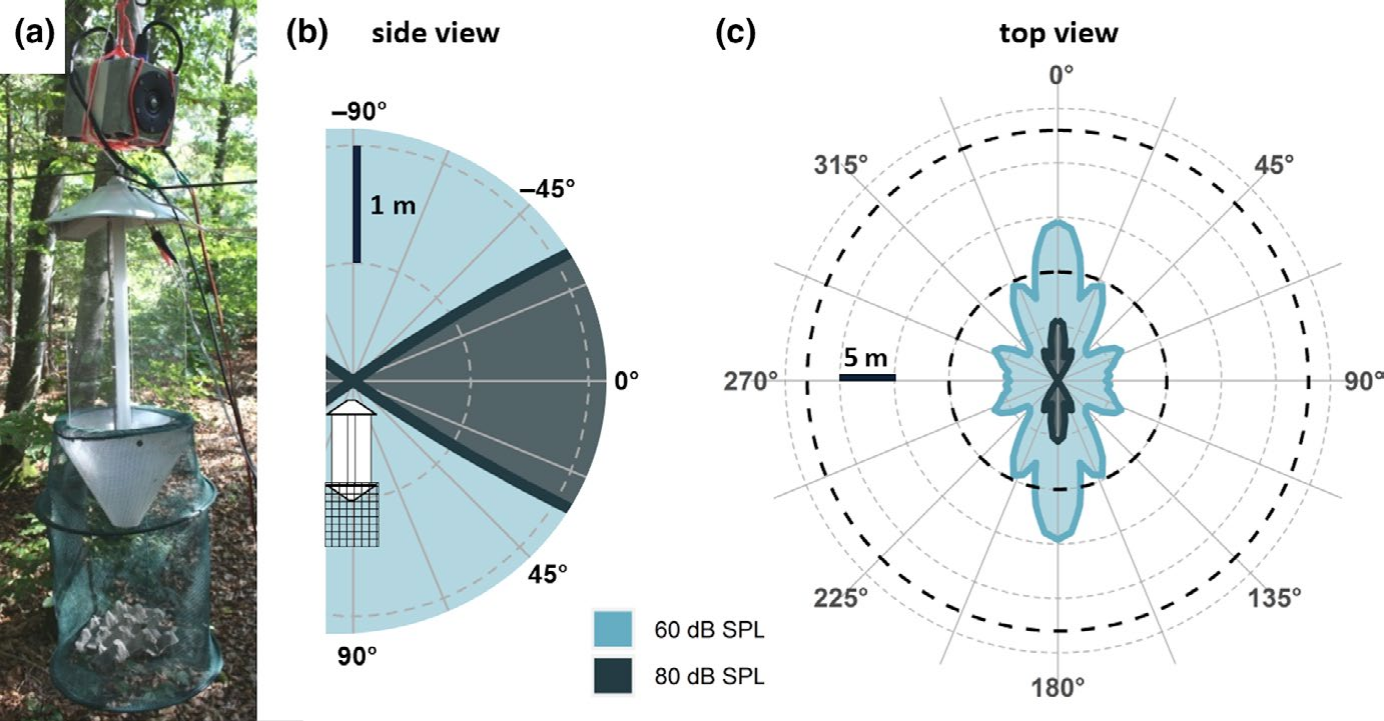
(a) Experimental setup. The photograph shows one of the light traps with two loudspeakers attached above the trap, both of which broadcast the acoustic stimulus in opposite directions. (b) Side view of the biologically relevant sound field around the light trap with attached loudspeakers. Colored areas indicate areas with minimum sound pressure levels of 60 and 80 dB SPL re. 20 µPa RMS, which are biologically relevant acoustic thresholds for eliciting negative phonotaxis and last‐ditch maneuvers, respectively, in eared moths. (c) Top view of the biologically relevant sound field (colored areas) and light field (dashed lines). Dashed lines indicate the distance over which 5% of released noctuid (10 m) and geometrid (23 m) moths are recaptured at the light trap, respectively (Merckx & Slade, 2014), which we used as first approximation of the range where light might interact with the moths’ sound‐evoked antipredator flight

We compared the number of moths caught at two light traps, one of which was equipped with a loudspeaker. We set up two equal light traps (Sylvania, blacklight, F15W/350BL‐T8, Shanghai, China; Figure 1a): one next to a path in a forest (trap A) and the other one at 30 m distance in the forest (trap B), close to the Max Planck Institute for Ornithology, Seewiesen, Germany. Both traps hung freely at ~1.7 m above ground, radiating light at 360° in the horizontal plane (Figure 1a). Between 19 July and 16 August 2018, we collected data over 15 rainless nights, somewhat increasing the number of nights sampled in similar previous studies (2–12 nights (Treat, 1962) and 6 nights (Agee & Webb, 1969)). Each test night, we alternatingly equipped one of the two traps with two ultrasonic loudspeakers (ScanSpeak, Avisoft Bioacoustics, Glienicke, Germany), both broadcasting an ultrasonic stimulus (see below) to simulate echolocating bats. The two loudspeakers were fixed back to back facing in opposite directions and were mounted above the respective light trap at ~2 m above ground. Thus, each trap was associated for 7–8 nights with ultrasound simulating an echolocating bat. Each test night, lights and acoustic playback were turned on in the evening (between 20:30 and 23:40 hours) and turned off the next morning (between 7:00 and 9:40 hours). We emptied the traps each morning and counted all moths of the three ear‐possessing families Noctuidae, Geometridae, and Erebidae. We measured each individuals’ body length along the middorsal line (from the head to the end of the abdomen), to correct for the fact that larger moths have more sensitive hearing than smaller moths (Surlykke et al., 1999; ter Hofstede et al., 2013). For those individuals whose body length could not be measured (e.g., due to missing abdomen, *N* = 137, 15.5%), we either used the mean value of the species or, if this was not possible (*N* = 1), the mean value of the family. For statistical analysis, we binned individuals into six categories of body length (1.0–3.0 cm, bin width 0.5 cm).

### 2.2 Ultrasonic playback stimulus design and evaluation

We simulated predation pressure by echolocating bats by repeatedly playing a short bat‐like ultrasonic pure tone pulse. Pulse frequency was 35 kHz, matching most moths’ best hearing threshold around 20–50 kHz (Noctuidae: ter Hofstede et al., 2013; Nakano et al., 2015; Erebidae: ter Hofstede et al., 2008; Pyralidae: Skals & Surlykke, 2000; Geometridae: Rydell et al., 1997; Surlykke et al., 1997; Sphingidae/Drepanidae: Nakano et al., 2015). Pulse duration was 10 ms including 2 ms linear rise and fall times, corresponding to the calls of European open space bats (Obrist et al., 2004; Skiba, 2014) and optimizing information transfer to the moths (Gordon & ter Hofstede, 2018). Pulse interval was 100 ms, matching the call interval of searching bats (60 – 200 ms, Holderied & von Helversen, 2003; Skiba, 2014). On‐axis sound pressure level (SPL) was 98 dB SPL re. 20 µPa RMS at 1‐m distance (see below for a detailed description of the sound field). This stimulus was presented continuously in a loop throughout the night via the loudspeakers using Avisoft‐RECORDER software (Avisoft Bioacoustics), a sound card with amplifier (Avisoft UltraSoundGate 116, Avisoft Bioacoustics) and a laptop computer.

To test the effect of our acoustic stimulus in darkness, without the potentially suppressing influence of light, we exposed free‐flying moths in a dark flight room (5.3 m × 3.5 × 3 m^3^) to the same acoustic stimulus. We caught moths at trap A over the course of four nights and tested them within 30 hr after capture. We placed moths on the ground of the flight room and recorded the flight paths of upward‐flying moths with an IR‐sensitive camera (Sony HDR‐CX560, Sony, Tokyo, Japan) under IR illumination (850 nm, Mini IR Illuminator TV6700, ABUS Security‐Center, Wetter, Germany). Using the same audio equipment as described above, we manually started the sound presentation when a moth flew in front of the speaker. We subsequently categorized the video‐recorded flight paths as “reaction” when the flight direction, level of erraticness, or both changed with stimulus onset (see supplementary video for examples); as “no reaction” when we did not observe those changes; or as “ambiguous” when we could not clearly assign the flight path to one of the two previous categories.

### 2.3 Overlap of sound and light field

The range and geometry of the presented light and sound fields differed. While the light was emitted omnidirectionally in the horizontal plane, the sound field was directional (Figure 1). We estimated the biologically relevant range for attracting moths by light and the biologically relevant sound fields for triggering moths’ evasive flight based on literature values and our own measurements.

Light traps can attract released moths over up to 80‐m distance, yet recaptures dramatically decrease beyond 15 m and depend on family (Merckx & Slade, 2014; Truxa & Fiedler, 2012). Family‐specific models estimated the 5%‐recapture rate at a distance of 10 ± 6 m (mean ± *SEM*) for Noctuidae and 23 ± 12 m for Geometridae (Merckx & Slade, 2014). Note, however, that these distances were obtained with a different light source than ours (6W actinic versus 15W blacklight in our case), and that the distance over which light attracts moths must not be equivalent to the distance over which light interferes with evasive flight. We still used these family‐specific distances as first approximation for the biologically relevant light fields where light might interact with the moths’ sound‐evoked antipredator flight (Figure 1c, dashed lines).

To estimate the effect of the playback, we measured the sound pressure level (SPL) of the played‐back pulse in front of the loudspeaker (on‐axis) and in steps of 5° up to 90° off‐axis (for details, see SI). The on‐axis source level at 1‐m distance was 97 dB SPL re. 20 µPa RMS. This is within the lower range of the call levels emitted by free‐flying bats, which is 100–120 dB peSPL @ 1 m (Goerlitz et al., 2020; Holderied & von Helversen, 2003), which corresponds to RMS‐SPL levels that are ~3–7 dB lower than the peSPL levels (Lewanzik & Goerlitz, 2017; Seibert et al., 2015). With increasing off‐axis angle, the source level became fainter by up to ~30 dB at 45°, resulting in a minimum playback level of 70 dB SPL RMS @ 1m. We then calculated the angle‐dependent distances around the loudspeaker where the playback would reach biologically relevant levels of 60 and 80 dB SPL RMS. We chose 60 and 80 dB SPL as approximate thresholds for eliciting negative phonotaxis and last‐ditch maneuvers, respectively, based on several lines of evidence. Negative phonotaxis and last‐ditch maneuvers are likely triggered at levels somewhat above the thresholds of the moths’ auditory receptor neurons A1 and A2, respectively (Gordon & ter Hofstede, 2018; Madsen & Miller, 1987; Roeder, 1974). The best thresholds of the A1 neuron are at about 35–55 dB SPL, and of the A2 neuron at about ~52–72 dB SPL (Gordon & ter Hofstede, 2018; ter Hofstede & Ratcliffe, 2016; Madsen & Miller, 1987; Surlykke, 2003; ter Hofstede et al., 2013; Waters & Jones, 1996). Behavioral thresholds in moths are largely unknown, but those that are known tend to be about 10 dB higher than neuronal thresholds (reviewed in Lewanzik & Goerlitz, 2017). We thus defined 60 and 80 dB SPL RMS as thresholds that will likely elicit negative phonotaxis and last‐ditch maneuvers in most moth species, respectively, and calculated their isolines of constant sound pressure levels. SPL isolines varied with the angle around the loudspeaker, ranging from 3.6 to 14.6 m for 60 dB SPL RMS, and from 0 to 5.6 m for 80 dB SPL RMS (Figure 1b&c). In summary, we thus presented a highly directional sound field in an omnidirectional light field. Thus, moths that approached the light trap in the on‐axis direction of the loudspeaker experienced gradually increasing SPLs sufficiently high to first elicit negative phonotaxis and later last‐ditch maneuvers. In contrast, moths that approached off‐axis from the loudspeaker experienced gradually increasing SPLs that were only sufficiently high to elicit negative phonotaxis, but not last‐ditch maneuvers.

### 2.4 Statistical analysis

To test for an effect of light on the moths’ evasive flight, we fitted linear models to the logarithmized moth count data as a function of the fixed effects *playback*, *trap*, *moth family*, and *moth body length,* and the interactions of *playback* and *moth family*, and of *playback* and *moth body length*.

We preferred this linear model with logarithmized count data over a negative bionomial model, even though both model types could be fitted similarly well, because the linear model enabled us to perform detailed power analysis. Although we have repeated measures over 15 nights, we did not include date as a random factor as it only explained a minor proportion of the variance in the logarithmized data.

We defined the moth family Noctuidae as intercept, as it had the largest sample size and thus was the most reliable reference. To test which factors significantly contributed to the model fit, we conducted backward model reduction (Lewis et al., 2011). Hence, the full model was successively reduced, by stepwise removing factors, starting with the factor having the highest p‐value of the t‐statistics provided by the model summary. We compared models with likelihood ratio tests using a *F*‐statistic and AICs. None of the interaction terms nor *trap* contributed significantly to model fit and were thus excluded. *Moth family* and *moth body length* contributed significantly to model fit (see results). Even though it did not contribute significantly to the model fit, we also kept *playback* as factor in the final model as this was the key parameter whose effect on the number of caught moths we aimed to analyze. Our final model thus included *playback*, *moth family,* and *moth body length* as fixed factors, without any interactions.

We evaluated the power of our model for the effect sizes found in the field and in the flight room by a randomization approach of our real dataset. We first randomized the factor playback and then added an effect size as determined in the flight room or in the field to those logarithmized moth counts where the playback was “on.” We ran this simulation 10,000 times for both effect sizes, and each time compared models (final model vs. final model without *playback*) with likelihood ratio tests using a *F*‐statistic to test for a significant effect of *playback* on the model fit. The proportion of significant effects of *playback* per 10,000 simulations equals our power to detect an effect of the tested effect size.

All statistical analyses were conducted in R version 3.3.2 (R Foundation for Statistical Computing, Vienna, Austria) using the packages “lme4”(Bates et al., 2015), “MASS” (Venables & Ripley, 2002), “blmeco” (Korner‐Nievergelt et al., 2015), “DHARMa” (Hartig, 2019), “car” (Fox, 2019), “effectsize” (Kassambara, 2019), and RNOmni” (McCaw, 2018) for statistics and the packages “ggplot2” (Wickham, 2020), “dplyr” (Wickham, 2018), “cowplot” (Wilke, 2019), and “ggthemer” (Arnold, 2018) for data sorting and plotting. For further details, see the R‐script provided with our data.

## 3 RESULTS

Of 33 moths tested in free‐flight in the dark flight room, ten moths (30.3%) showed an evasive reaction in response to the acoustic stimulus. Twelve moths (36.4%) did not react, and eleven moths (33.3%) were categorized as “ambiguous.” The moths’ distance to the speaker at stimulus onset was about 1–2 m. Our acoustic stimulus thus was audible and elicited an evasive reaction in about one third of the tested moths (see supplementary video for examples). When considering the ambiguous reactions, the stimulus might even be audible to a larger proportion of up to two thirds of the moth population.

**Figure 2:**
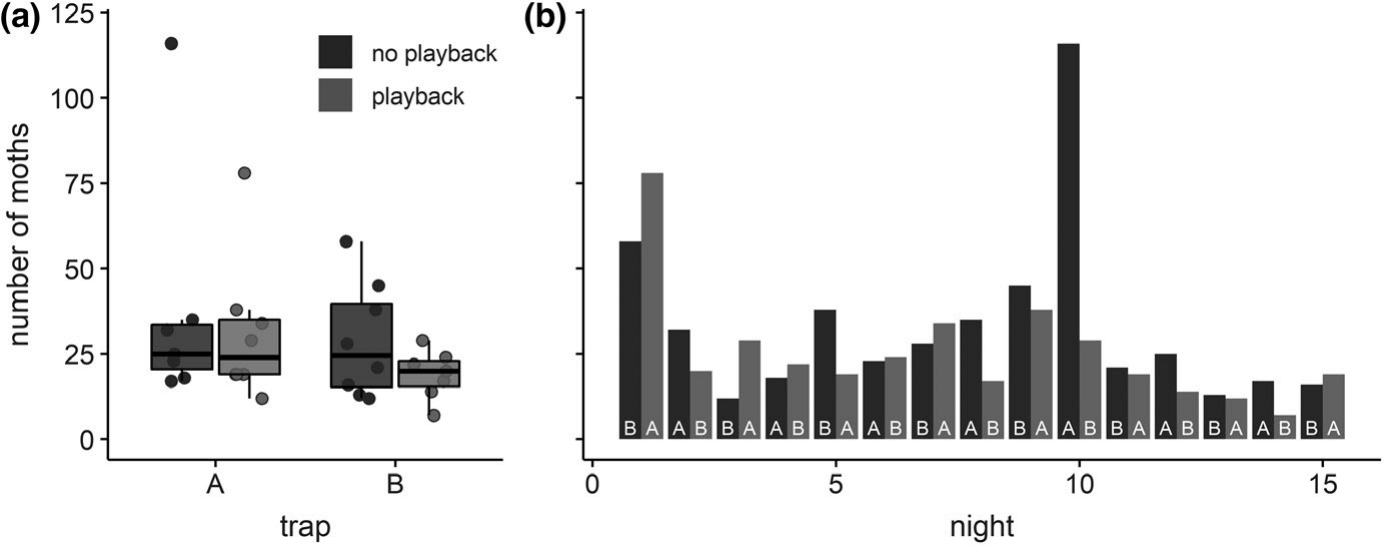
Number of caught moths per playback treatment, per trap and per night. (a) Number of caught moths at both light traps, with and without playback. Boxplots show median, quartiles, and whiskers (up to 1.5 × interquartile range beyond the quartiles) of the daily counts (dots). (b) Daily counts of caught moths per night and per trap for all 15 nights. White letters at the base of the bars indicate the trap

In the field experiment, we caught a total of 878 moths over 15 nights, with a median of ~23 moths caught per night and light trap (Figure 2a), yet with large fluctuations between nights and smaller fluctuations between traps (7–116 moths per night and trap; Figure 2b). We mostly caught moths of the family Noctuidae (80.9%), followed by Geometridae (11.1%) and Erebidae (7.8%), and we caught more smaller than larger moths. Accordingly, both *moth family* and *moth body length* contributed significantly to our final model after stepwise model reduction (likelihood ratio tests: *moth family*, *F*_(153,2)_ = 31.92, *p* < .001; *moth body length*, *F*_(153,3)_ = 34.43, *p* < .001).

Although the total number of moths caught at trap A (506 moths) was 41% higher than the total number caught at trap B (365 moths), this was largely driven by one night (night 10:115 vs. 29 moths; Figure 2b). Across all 15 nights, the nightly capture rate did not significantly differ between traps (likelihood ratio test for factor *trap*: *F*_(151,1)_ = 0.53, *p* = .468; Figure 2a).

We could not detect an effect of our acoustic playback on the nightly capture rates (Figure 2a; modeled effect size of factor *playback* on logarithmized moth count at the playback trap relative to the silent trap: −0.06 (95% CI: −0.16 to + 0.04), corresponding to 86% (95% CI: 69%–109%) capture rate at the playback trap relative to the silent trap; likelihood ratio test of factor *playback*, *F*_(152,1)_ = 1.53, *p* = .218).

We analyzed the power of our experiment, both for the effect size observed in the field (−0.06) and in the flight room. In the flight room, at least 30% of the moths reacted to our acoustic playback. Assuming that those reacting moths would not be caught in the light trap, the capture rate at the ultrasonic trap would be 70% relative to the silent trap, resulting in an expected effect size of the playback of −0.16 (in logarithmized moth counts). The power is the probability of rejecting the null hypothesis (i.e., obtaining a statistically significant result at a chosen significance level), given that the null hypothesis is false. Based on our dataset and sample size, our field experiment had a very low power of only 21% to detect an effect as small as the one observed in the field. In contrast, we had a sufficiently high power of 87% to detect an effect as large as the one observed in the flight room. This suggests that the lack of a significant effect of *playback* in the field might have been caused by a light‐induced decrease in the effect size compared to the dark flight room—that is, a light‐induced suppression of sound‐evoked antipredator flight.

## 4 DISCUSSION

Echolocating bats and eared insects are a textbook example of sound‐mediated predator–prey interaction (ter Hofstede & Ratcliffe, 2016). Increasing light pollution (Fouquet, 2006; Hölker et al., 2010), however, severely impacts both bats and moths (Cravens et al., 2018; Macgregor et al., 2019; Stone et al., 2009), with potential cascading effects on their predator–prey interactions, population dynamics, and ecosystems (Minnaar et al., 2015; Russo et al., 2019). While good evidence exists that light reduces the sound‐evoked last‐ditch maneuvers of eared moths, the effect of light on the moth's first line of defense, negative phonotaxis, and thus the overall effect of light on moth antipredator flight, is unclear. Here, we compared moth captures at two light traps. One trap was silent, while the other trap broadcast bat‐like ultrasonic stimuli aimed to trigger last‐ditch maneuvers and negative phonotaxis. We did not find a significant reduction in the number of caught moths at the ultrasonic light trap. There is, however, a high bar for showing that a treatment (such as our ultrasonic playback) has no effect. The power of our field experiment was too low to detect significant changes in moth count at an effect size as small as we observed in the field. In contrast, our field experiment had sufficient power to detect a potential effect of the playback with an effect size as large as observed in the dark flight room, if this effect had been present under lit field conditions. One conclusion thus is that the light suppressed both types of the moths’ sound‐evoked antipredator flight, negative phonotaxis and last‐ditch maneuvers.

There are, however, alternative explanations in addition to a light‐induced suppression of antipredator flight. The field and flight room experiments differed not only in the light level, but also in temporal (full night vs. short‐term sound exposure) and spatial (variable distances between moth and loudspeaker vs. close‐range to the loudspeaker) parameters. In addition, physiological and behavioral states of the moths will likely differ between free‐flying moths in the field and captured and released moths in the flight room.

The continuous ultrasonic playback over a full night might cause the moths to habituate to the playback. Habituation was previously suggested as an explanation for playback‐independent capture rates of male *Helicoverpa zea* moths at pheromone traps (Gillam et al 2011), although habituation was not shown at the neuronal level in response to searching bats (Gordon & ter Hofstede, 2018). However, although our playback was on throughout the night, the extent to which a given individual moth was exposed to the playback depends on the speed and trajectory of its own flight behavior. Arguably, our experimental situation might be similar to the realistic case of moths at street lights that are attacked by close‐by bats (Rydell, 1992). Indeed, Treat (1962) and Agee and Webb (1969), whose studies our study was based on, also presented ultrasonic playbacks throughout the night and did detect differences between the silent and ultrasonic trap, which argues against habituation. We also believe that differences in stimulus design cannot explain the differences between experiments. Treat (1962) and Agee and Webb (1969) broadcast multiple stimuli varying in pulse rate, frequency, duration, and sound pressure level (ranges: 0.7–155 pulses/s; 12.5–200 kHz; 2–10 ms; SPL: ~60–100 dB SPL @ 1‐m distance), all of which elicited varying degrees of evasive flight in eared moths. Our stimulus had acoustic properties within this range and did elicit evasive flight under dark control conditions, yet seems not to elicit sufficient evasive flight under lit conditions.

When assuming that light indeed suppressed the moths’ sound‐evoked antipredator flight in our experiment, the question arises why this was not the case in the similar light‐trap experiments by Treat (1962) and Agee and Webb (1969). We propose that the differences in the geometry and overlap of light and sound fields might explain these contrasting results. In the previous setups (Agee & Webb, 1969; Treat, 1962), sound and light fields almost overlapped and were emitted within a relatively narrow angle. Before entering the light trap, approaching moths thus passed through high sound pressure levels that would likely elicit last‐ditch maneuvers. As both studies caught fewer moths in the ultrasonic trap than the silent traps, this suggests that the playback still elicited some antipredator flight (likely last‐ditch maneuvers) despite the light. Specifically, for stimuli similar to ours, the relative capture rates between the ultrasonic and silent traps were 35% versus 65% for noctuid moths and 37.5 kHz tone pulses (Treat, 1962), and 15% versus 85% for two species of the families Noctuidae and Pyralidae and 30 kHz tone pulses (Agee & Webb, 1969). In contrast, our setup combined an omnidirectional light field with a directional sound field, thus exposing the moths to different sound pressure levels (SPL) depending on approach direction. When approaching the trap on‐axis of the loudspeaker's main axis, received SPLs increased from low to high, which should first elicit negative phonotaxis and later last‐ditch maneuvers. In contrast, when approaching the traps off‐axis, SPLs remained so low to only elicit negative phonotaxis. As our capture rates did not differ among the light traps—indicating that the playback did not evoke antipredator flight—we suggest that the light not only reduced last‐ditch behavior, but also the negative phonotaxis of eared moths. Even if the moths still exhibited some degree of last‐ditch maneuver close to the trap (as shown by Treat, 1962 and Agee & Webb, 1969), these maneuvers might have brought the moth into a position off‐axis to the loudspeaker with low SPL (either to the side or below the loudspeaker's main axis). From there, no further last‐ditch maneuvers would have been elicited due to the low SPL, while the light kept attracting the moth into the trap and suppressed negative phonotaxis. Our results are in line with those of Minnaar et al. (2015), who found that a model of escape behavior in moths assuming 0% escape efficacy best explained bats’ diet under lit conditions. We therefore suggest that light suppresses not only last‐ditch maneuvers, as previously shown, but also negative phonotaxis. Further experiments need to validate this suggestion, by testing low and high source levels separately (either separated spatially or temporally), by increasing the number of nightly and spatial replicates, and by testing additional bat‐like sounds for a variety of light and sound level combinations. In addition, tracking and quantification of the three‐dimensional evasive flight of individual moths of different species will provide detaile insights into the variability of their evasive flight, and the effect of light on it.

In summary, our results underline the strong effect of light on eared moths and suggest that both types of antipredator flight are suppressed by light. It is important to note, though, that our study design tested the effect of light only indirectly, by testing the effect of sound on light‐mediated moth captures, not the effect of light on sound‐mediated evasive flight. In addition, showing no effect is difficult and is complicated by the natural variability of moth behavior (Hügel & Goerlitz 2019), potentially complicating simple answers. If increasing artificial light at night suppresses both negative phonotaxis and last‐ditch maneuvers, moths are not only unable to escape nearby predators, but also unable to avoid distant predators by flying away. Similarly, fish are attracted to lit areas, where they are “trapped” and preyed upon by seals (Yurk & Trites, 2000) and other fish (Becker et al., 2013). The increasing levels of light pollution demand for further studies to understand the mechanism(s) of how light attracts animals and interferes with their behavior.

## CONFLICT OF INTEREST

We have no competing interests.

## AUTHOR CONTRIBUTION

**Theresa Hügel:** Conceptualization (equal); Data curation (lead); Formal analysis (lead); Funding acquisition (supporting); Investigation (lead); Methodology (lead); Project administration (equal); Software (lead); Validation (lead); Visualization (lead); Writing‐original draft (lead); Writing‐review & editing (supporting). **Holger Goerlitz:** Conceptualization (equal); Formal analysis (supporting); Funding acquisition (lead); Investigation (supporting); Methodology (supporting); Project administration (equal); Supervision (lead); Validation (supporting); Visualization (supporting); Writing‐original draft (supporting); Writing‐review & editing (lead).

## Ethics Approval

Permission to carry out fieldwork was provided by the relevant German authority (Landratsamt Starnberg, permit # 55.1‐8646.NAT_02‐8‐41).

## Supporting information

Supplementary Material

Video S1

## Data Availability

All data and R‐scripts are provided at Dryad at 10.5061/dryad.mcvdncjzqwith (https://doi.org/10.5061/dryad.mcvdncjzq).

## References

[ece36904-bib-0001] Acharya, L. , & Fenton, M. B. (1999). Bat attacks and moth defensive behaviour around street lights. Canadian Journal of Zoology, 77(1), 27–33. 10.1139/z98-202

[ece36904-bib-0002] Agee, H. R. (1969). Response of flying bollworm moths and other tympanate moths to pulsed ultrasound. Annals of the Entomological Society of America, 62(4), 801–807. 10.1093/aesa/62.4.801

[ece36904-bib-0003] Agee, H. R. , & Webb, J. C. (1969). Effects of ultrasound on capture of *Heliothis zea* and *Ostrinia nubilalis* moths in traps equipped with ultraviolet lamps. Annals of the Entomological Society of America, 62(6), 1248–1252. 10.1093/aesa/62.6.1248 5374163

[ece36904-bib-0004] Arnold, J. B. (2018). Ggthemes Package | R Documentation. https://www.rdocumentation.org/packages/ggthemes/versions/3.5.0

[ece36904-bib-0005] Bailey, L. A. , Brigham, R. M. , Bohn, S. J. , Boyles, J. G. , & Smit, B. (2019). An experimental test of the allotonic frequency hypothesis to isolate the effects of light pollution on bat prey selection. Oecologia, 190(2), 367–374. 10.1007/s00442-019-04417-w 31139944

[ece36904-bib-0006] Bates, D. , Maechler, M. , Bolker, B. , & Walker, S. (2015). Fitting linear mixed‐effects models using lme4. Journal of Statistical Software, 67(1), 1–48. 10.18637/jss.v067.i01

[ece36904-bib-0007] Becker, A. , Whitfield, A. K. , Cowley, P. D. , Järnegren, J. , & Næsje, T. F. (2013). Potential effects of artificial light associated with anthropogenic infrastructure on the abundance and foraging behaviour of estuary‐associated fishes. Journal of Applied Ecology, 50(1), 43–50. 10.1111/1365-2664.12024

[ece36904-bib-0008] Boyles, J. G. , Cryan, P. M. , McCracken, G. F. , & Kunz, T. H. (2011). Economic importance of bats in agriculture. Science, 332(6025), 41–42. 10.1126/science.1201366 21454775

[ece36904-bib-0009] Cravens, Z. M. , Brown, V. A. , Divoll, T. J. , & Boyles, J. G. (2018). Illuminating prey selection in an insectivorous bat community exposed to artificial light at night. Journal of Applied Ecology, 55(2), 705–713. 10.1111/1365-2664.13036

[ece36904-bib-0010] Davies, T. W. , Bennie, J. , Inger, R. , Ibarra, N. H. , & Gaston, K. J. (2013). Artificial light pollution: Are shifting spectral signatures changing the balance of species interactions? Global Change Biology, 19(5), 1417–1423. 10.1111/gcb.12166 23505141 PMC3657119

[ece36904-bib-0011] Davies, T. W. , Duffy, J. P. , Bennie, J. , & Gaston, K. J. (2014). The nature, extent, and ecological implications of marine light pollution. Frontiers in Ecology and the Environment, 12(6), 347–355. 10.1890/130281

[ece36904-bib-0012] Davies, T. W. , & Smyth, T. (2018). Why artificial light at night should be a focus for global change research in the 21st century. Global Change Biology, 24(3), 872–882. 10.1111/gcb.13927 29124824

[ece36904-bib-0013] Falchi, F. , Cinzano, P. , Duriscoe, D. , Kyba, C. C. M. , Elvidge, C. D. , Baugh, K. , Portnov, B. A. , Rybnikova, N. A. , & Furgoni, R. (2016). The new world atlas of artificial night sky brightness. Science Advances, 2(6), e1600377 10.1126/sciadv.1600377 27386582 PMC4928945

[ece36904-bib-0014] Fenton, M. B. (2003). Eavesdropping on the echolocation and social calls of bats. Mammal Review, 33, 193–204. 10.1046/j.1365-2907.2003.00019.x

[ece36904-bib-0015] Fouquet, R. (2006). Seven centuries of energy services: The price and use of light in the United Kingdom (1300–2000). Energy Journal, 27, 139–177. 10.2307/23296980

[ece36904-bib-0016] Fox, J. (2019). Car Package | R Documentation. https://www.rdocumentation.org/packages/car/versions/3.0‐9

[ece36904-bib-0017] Goerlitz, H. R. , ter Hofstede, H. M. , & Holderied, M. W. (2020). Neural representation of bat predation risk and evasive flight in moths: A modelling approach. Journal of Theoretical Biology, 486, 110082 10.1016/j.jtbi.2019.110082 31734242

[ece36904-bib-0018] Gordon, S. D. , & ter Hofstede, H. M. (2018). The influence of bat echolocation call duration and timing on auditory encoding of predator distance in noctuoid moths. Journal of Experimental Biology, 221(6). 10.1242/jeb.171561 29567831

[ece36904-bib-0019] Hartig, F. (2019). DHARMa: Residual Diagnostics for Hierarchical (Multi‐Level / Mixed) Regression Models. http://florianhartig.github.io/DHARMa/

[ece36904-bib-0020] Holderied, M. W. , & von Helversen, O. (2003). Echolocation range and wingbeat period match in aerial‐hawking bats. Proceedings of the Royal Society of London. Series B: Biological Sciences, 270, 2293–2299. 10.1098/rspb.2003.2487 14613617 PMC1691500

[ece36904-bib-0021] Hölker, F. , Moss, T. , Griefahn, B. , Kloas, W. , Voigt, C. C. , Henckel, D. , Hänel, A. , Kappeler, P. M. , Völker, S. , Schwope, A. , Franke, S. , Uhrlandt, D. , Fischer, J. , Klenke, R. , Wolter, C. , & Tockner, K. (2010). The dark side of light: A transdisciplinary research agenda for light pollution policy. Ecology and Society, 15(4). 10.5751/ES-03685-150413

[ece36904-bib-0022] Hügel, T. , & Goerlitz, H. R. (2019). Species‐specific strategies increase unpredictability of escape flight in eared moths. Functional Ecology, 33(9), 1674–1683.

[ece36904-bib-0023] Kassambara, A. (2019). Rstatix Package | R Documentation. https://www.rdocumentation.org/packages/rstatix/versions/0.6.0

[ece36904-bib-0024] Kasso, M. , & Balakrishnan, M. (2013). Ecological and economic importance of bats (order Chiroptera). ISRN Biodiversity , 2013, 1–9. 10.1155/2013/187415

[ece36904-bib-0025] Knop, E. , Zoller, L. , Ryser, R. , Gerpe, C. , Hörler, M. , & Fontaine, C. (2017). Artificial light at night as a new threat to pollination. Nature, 548(7666), 206–209. 10.1038/nature23288 28783730

[ece36904-bib-0026] Korner‐Nievergelt, F. , Roth, T. , von Felten, S. , Guelat, J. , Almasi, B. , & Korner‐Nievergelt, P. (2015). Blmeco. https://www.rdocumentation.org/packages/blmeco/versions/1.4

[ece36904-bib-0027] Kunz, T. H. , Braun de Torrez, E. , Bauer, D. , Lobova, T. , & Flemming, T. H. (2011). Ecosystem services provided by bats. Annals of the New York Academy of Sciences, 1223(1), 1–38. 10.1111/j.1749-6632.2011.06004.x 21449963

[ece36904-bib-0028] Lewanzik, D. , & Goerlitz, H. R. (2017). Continued source level reduction during attack in the low‐amplitude bat *Barbastella barbastellus* prevents moth evasive flight. Functional Ecology, 32(5), 1251–1261. 10.1111/1365-2435.13073

[ece36904-bib-0029] Lewis, F. , Butler, A. , & Gilbert, L. (2011). A unified approach to model selection using the likelihood ratio test. Methods in Ecology and Evolution, 2(2), 155–162. 10.1111/j.2041-210X.2010.00063.x

[ece36904-bib-0030] Longcore, T. , & Rich, C. (2004). Ecological light pollution. Frontiers in Ecology and the Environment, 2(4), 191–198. 10.1890/1540-9295(2004)002[0191:ELP]2.0.CO;2

[ece36904-bib-0031] Macgregor, C. J. , Evans, D. M. , Fox, R. , & Pocock, M. J. O. (2017). The dark side of street lighting: Impacts on moths and evidence for the disruption of nocturnal pollen transport. Global Change Biology, 23(2), 697–707. 10.1111/gcb.13371 27251575

[ece36904-bib-0032] Macgregor, C. J. , Pocock, M. J. O. , Fox, R. , & Evans, D. M. (2019). Effects of street lighting technologies on the success and quality of pollination in a nocturnally pollinated plant. Ecosphere, 10(1), e02550 10.1002/ecs2.2550

[ece36904-bib-0033] Madsen, B. M. , & Miller, L. A. (1987). Auditory input to motor neurons of the dorsal longitudinal flight muscles in a noctuid moth (*Barathra brassicae L*.). Journal of Comparative Physiology A, 160(1), 23–31. 10.1007/BF00613438

[ece36904-bib-0034] Mathews, F. , Roche, N. , Aughney, T. , Jones, N. , Day, J. , Baker, J. , & Langton, S. (2015). Barriers and benefits: Implications of artificial night‐lighting for the distribution of common bats in Britain and Ireland. Philosophical Transactions of the Royal Society of London. Series B, Biological Sciences, 370(1667), 20140124 10.1098/rstb.2014.0124 25780236 PMC4375364

[ece36904-bib-0035] McCaw, Z. (2018). RNOmni Package | R Documentation. https://www.rdocumentation.org/packages/RNOmni/versions/0.3.0

[ece36904-bib-0036] Merckx, T. , & Slade, E. M. (2014). Macro‐moth families differ in their attraction to light: Implications for light‐trap monitoring programmes. Insect Conservation and Diversity, 7(5), 453–461. 10.1111/icad.12068

[ece36904-bib-0037] Miller, C. R. , Barton, B. T. , Likai, Z. , Radeloff, V. C. , Oliver, K. M. , Harmon, J. P. , & Ives, A. R. (2017). Combined effects of night warming and light pollution on predator–prey interactions. Proceedings of the Royal Society B: Biological Sciences, 284(1864), 20171195 10.1098/rspb.2017.1195 PMC5647293 29021171

[ece36904-bib-0038] Minnaar, C. , Boyles, J. G. , Minnaar, I. A. , Sole, C. L. , & McKechnie, A. E. (2015). Stacking the odds: Light pollution may shift the balance in an ancient predator‐prey arms race. Journal of Applied Ecology, 52(2), 522–531. 10.1111/1365-2664.12381

[ece36904-bib-0039] Nakano, R. , Takanashi, T. , & Surlykke, A. (2015). Moth hearing and sound communication. Journal of Comparative Physiology A, 201(1), 111–121. 10.1007/s00359-014-0945-8 25261361

[ece36904-bib-0040] Obrist, M. K. , Boesch, R. , & Fluckiger, P. F. (2004). Variability in echolocation call design of 26 Swiss bat species: Consequences, limits and options for automated field identification with a synergetic pattern recognition approach. Mammalia, 68, 307–322. 10.1515/mamm.2004.030

[ece36904-bib-0041] Owens, A. C. S. , & Lewis, S. M. (2018). The impact of artificial light at night on nocturnal insects: A review and synthesis. Ecology and Evolution, 8(22), 11337–11358. 10.1002/ece3.4557 30519447 PMC6262936

[ece36904-bib-0042] Roeder, K. D. (1962). The behaviour of free flying moths in the presence of artificial ultrasonic pulses. Animal Behavior, 10(3–4), 300–304. 10.1016/0003-3472(62)90053-2

[ece36904-bib-0043] Roeder, K. D. (1964). Aspects of the noctuid tympanic nerve response having significance in the avoidance of bats. Journal of Insect Physiology, 10(4), 529–546. 10.1016/0022-1910(64)90025-3

[ece36904-bib-0044] Roeder, K. D. (1967). Turning tendency of moths exposed to ultrasound while in stationary flight. Journal of Insect Physiology, 13(6), 873–888. 10.1016/0022-1910(67)90051-0

[ece36904-bib-0045] Roeder, K. D. (1974). Responses of the less sensitive acoustic sense cells in the tympanic organs of some noctuid and geometrid moths. Journal of Insect Physiology, 20(1), 55–66. 10.1016/0022-1910(74)90123-1 4811211

[ece36904-bib-0046] Russo, D. , Cosentino, F. , Festa, F. , De Benedetta, F. , Pejic, B. , Cerretti, P. , & Ancillotto, L. (2019). Artificial illumination near rivers may alter bat‐insect trophic interactions. Environmental Pollution, 252, 1671–1677. 10.1016/j.envpol.2019.06.105 31284209

[ece36904-bib-0047] Rydell, J. (1992). Exploitation of insects around streetlamps by bats in Sweden. Functional Ecology, 6(6), 744–750. 10.2307/2389972

[ece36904-bib-0048] Rydell, J. , Skals, N. , Surlykke, A. , & Svensson, A. M. (1997). Hearing and bat defence in geometrid winter moths. Proceedings of the Royal Society of London. Series B: Biological Sciences, 264(1378), 83–88. 10.1098/rspb.1997.0012 9061963 PMC1688226

[ece36904-bib-0049] Schnitzler, H.‐U. , Moss, C. F. , & Denzinger, A. (2003). From spatial orientation to food acquisition in echolocating bats. Trends in Ecology & Evolution, 18, 386–394. 10.1016/S0169-5347(03)00185-X

[ece36904-bib-0050] Seibert, A.‐M. , Koblitz, J. C. , Denzinger, A. , & Schnitzler, H.‐U. (2015). Bidirectional echolocation in the bat *Barbastella barbastellus*: Different signals of low source level are emitted upward through the nose and downward through the mouth. PLoS One, 10(9), e0135590 10.1371/journal.pone.0135590 26352271 PMC4564259

[ece36904-bib-0051] Skals, N. , & Surlykke, A. (2000). Hearing and evasive behaviour in the greater wax moth, *Galleria mellonella* (Pyralidae). Physiological Entomology, 25(4), 354–362. 10.1111/j.1365-3032.2000.00204.x

[ece36904-bib-0052] Skiba, R. (2014). Europäische Fledermäuse, 2nd ed VerlagsKG Wolf.

[ece36904-bib-0053] Stone, E. L. , Harris, S. , & Jones, G. (2015). Impacts of artificial lighting on bats: A review of challenges and solutions. Mammalian Biology, 80(3), 213–219. 10.1016/j.mambio.2015.02.004

[ece36904-bib-0054] Stone, E. L. , Jones, G. , & Harris, S. (2009). Street lighting disturbs commuting bats. Current Biology, 19(13), 1123–1127. 10.1016/j.cub.2009.05.058 19540116

[ece36904-bib-0055] Straka, T. M. , Greif, S. , Schultz, S. , Goerlitz, H. R. , & Voigt, C. C. (2020). The effect of cave illumination on bats. Global Ecology and Conservation, 21, e00808 10.1016/j.gecco.2019.e00808

[ece36904-bib-0056] Straka, T. M. , Lentini, P. E. , Lumsden, L. F. , Wintle, B. A. , & van der Ree, R. (2016). Urban bat communities are affected by wetland size, quality, and pollution levels. Ecology and Evolution, 6(14), 4761–4774. 10.1002/ece3.2224 27547311 PMC4979705

[ece36904-bib-0057] Surlykke, A. (2003). Hearing in hooktip moths (Drepanidae: Lepidoptera). Journal of Experimental Biology, 206(15), 2653–2663. 10.1242/jeb.00469 12819271

[ece36904-bib-0058] Surlykke, A. , Filskov, M. , Fullard, J. H. , & Forrest, E. (1999). Auditory relationships to size in noctuid moths: Bigger is better. Naturwissenschaften, 86(5), 238–241. 10.1007/s001140050607f

[ece36904-bib-0059] Surlykke, A. , Filskov, M. , Sanderson, C. G. , Lal, D. , Peters, B. , & Winkler, R. (1997). Hearing in geometrid moths. Naturwissenschaften, 84, 356–359. 10.1007/s001140050410

[ece36904-bib-0060] Svensson, A. M. A. , & Rydell, J. (1998). Mercury vapour lamps interfere with the bat defence of tympanate moths (Operophtera spp.; Geometridae). Animal Behaviour, 55(1), 223–226. 10.1006/anbe.1997.0590 9480689

[ece36904-bib-0061] Ter Hofstede, H. M. , Goerlitz, H. R. , Ratcliffe, J. M. , Holderied, M. W. , & Surlykke, A. (2013). The simple ears of noctuoid moths are tuned to the calls of their sympatric bat community. Journal of Experimental Biology, 216(21), 3954–3962. 10.1242/jeb.093294 23913945

[ece36904-bib-0062] ter Hofstede, H. M. , & Ratcliffe, J. M. (2016). Evolutionary escalation: The bat‐moth arms race. Journal of Experimental Biology, 219, 1589–1602. 10.1242/jeb.086686 27252453

[ece36904-bib-0063] ter Hofstede, H. M. , Ratcliffe, J. M. , & Fullard, J. H. (2008). Nocturnal activity positively correlated with auditory sensitivity in noctuoid moths. Biology Letters, 4(3), 262–265. 10.1098/rsbl.2007.0617 18319206 PMC2610036

[ece36904-bib-0064] Treat, A. E. (1962). Comparative moth catches by an ultrasonic and a silent light trap. Annals of the Entomological Society of America, 55(6), 716–720. 10.1093/aesa/55.6.716

[ece36904-bib-0065] Truxa, C. , & Fiedler, K. (2012). Attraction to light ‐ from how far do moths (Lepidoptera) return to weak artificial sources of light? Eur J Entomol, 109(1), 77–84. 10.14411/eje.2012.010

[ece36904-bib-0066] van Langevelde, F. , van Grunsven, R. H. A. , Veenendaal, E. M. , & Fijen, T. P. M. (2017). Artificial night lighting inhibits feeding in moths. Biology Letters, 13(3), 20160874 10.1098/rsbl.2016.0874 28250209 PMC5377031

[ece36904-bib-0067] Van Toor, M. L. , O’Mara, M. T. , Abedi‐Lartey, M. , Wikelski, M. , Fahr, J. , & Dechmann, D. K. N. (2019). Linking colony size with quantitative estimates of ecosystem services of African fruit bats. Current Biology, 29(7), R237–R238. 10.1016/j.cub.2019.02.033 30939302

[ece36904-bib-0068] Venables, W. N. , & Ripley, B. D. (2002). Modern applied statistics with S, 4th ed Springer.

[ece36904-bib-0069] Wakefield, A. , Jones, G. , Stone, E. L. , Harris, S. , Jones, G. , & Harris, S. (2015). Light‐emitting diode street lights reduce last‐ditch evasive manoeuvres by moths to bat echolocation calls. Royal Society Open Science, 2(8), 150291 10.1098/rsos.150291 26361558 PMC4555863

[ece36904-bib-0070] Waters, D. A. , & Jones, G. (1996). The peripheral auditory characteristics of noctuid moths: Responses to the search‐phase echolocation calls of bats. Journal of Experimental Biology, 199(4), 847–856. 9318627 10.1242/jeb.199.4.847

[ece36904-bib-0071] Wickham, H. (2018). Dplyr Package | R Documentation. https://www.rdocumentation.org/packages/dplyr/versions/0.7.8

[ece36904-bib-0072] Wickham, H. (2020). Ggplot2 | R Documentation. https://www.rdocumentation.org/packages/ggplot2/versions/3.2.1

[ece36904-bib-0073] Wilke, C. (2019). Cowplot Package | R Documentation. https://www.rdocumentation.org/packages/cowplot/versions/1.0.0

[ece36904-bib-0074] Wilson, J. F. , Baker, D. , Cheney, J. , Cook, M. , Ellis, M. , Freestone, R. , Gardner, D. , Geen, G. , Hemming, R. , Hodgers, D. , Howarth, S. , Jupp, A. , Lowe, N. , Orridge, S. , Shaw, M. , Smith, B. , Turner, A. , & Young, H. (2018). A role for artificial night‐time lighting in long‐term changes in populations of 100 widespread macro‐moths in UK and Ireland: A citizen‐science study. Journal of Insect Conservation, 22(2), 189–196. 10.1007/s10841-018-0052-1

[ece36904-bib-0075] Yurk, H. , & Trites, A. W. (2000). Experimental attempts to reduce predation by harbor seals on out‐migrating juvenile salmonids. Transactions of the American Fisheries Society, 129(6), 1360–1366. 10.1577/1548-8659(2000)129<1360:EATRPB>2.0.CO;2

