# Supplementary material for: Light might suppress both types of sound‐evoked antipredator flight in moths
Source: Ecol Evol. 2020 Nov 2;10(23):13134–42. doi: 10.1002/ece3.6904 (PMC7713931; doi:10.1002/ece3.6904)
Supplement: Supplementary file 1 — Supplementary Material [file ECE3-10-13134-s001.pdf]

Light might suppress both types of sound-evoked anti-predator flight in moths

Theresa Hügel[1], Holger R. Goerlitz[1]

[1] Acoustic and Functional Ecology, Max Planck Institute for Ornithology, Seewiesen, Germany

Supplementary Information

Measurements and calculations for the sound fields of 60 and 80 dB SPL RMS

We measured the emitted sound pressure levels (SPL) at three different distances (1, 2, and 4 m; twice per distance) and at all angles from 0° (on-axis) to 90° off-axis in steps of 5° around the speaker (Fig. S1). Next, we corrected the measurements taken at 2 and 4 m distance for atmospheric attenuation (21.4°C, 42% rel. humidity) and geometric attenuation due to spherical spreading and back-calculated them to the SPLs at 1 m distance. Including the original measured values at 1 m distance, this resulted in 3-6 values for 1 m distance per angle (missing values due to clipping). We next calculated the mean of all values at 1 m distance per angle to obtain the angle-dependent sound pressure levels. These mean values were used as angle-depended source level of the loudspeakers to calculate the sound field around the loudspeaker (with atmospheric attenuation for 21.4°C and 42% rel. humidity and spherical spreading). Resulting sound fields can be seen in 1 B & C.

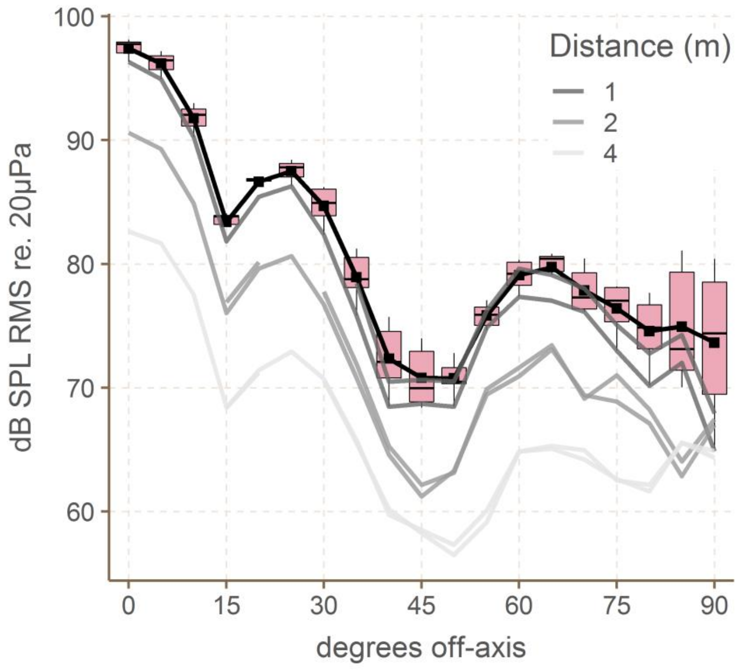

Figure S1: Measurement of the sound pressure level (SPL) around the loudspeaker. Measurements were taken at three distances (1,2 and 4 m) and angles from 0° to 90° in steps of 5°. Lines show the original measurements at all three distances, once measured from 0-90 degrees and once measured from 90-0 degrees. Missing values are due to clipping. Boxplots show the SPL at 1 m distance, combined from the original measurements at 1 m distance and back-calculated from the measurements taken at 2 and 4 m distance, indicating median, quartiles, whiskers (up to 1.5x the inter-quartile range beyond the quartiles). Black squares show means, which are connected by the black line.

Video S1: Examples of moths reacting to the ultrasonic playback in a dark flight room.

See the video provided as separate file in the online supporting information.
